# Progress in Discovering Transcriptional Noise in Aging

**DOI:** 10.3390/ijms24043701

**Published:** 2023-02-12

**Authors:** Josh Bartz, Hannim Jung, Karen Wasiluk, Lei Zhang, Xiao Dong

**Affiliations:** 1 Institute on the Biology of Aging and Metabolism, University of Minnesota, Minneapolis, MN 55455, USA; 2 Department of Genetics, Cell Biology and Development, University of Minnesota, Minneapolis, MN 55455, USA; 3 Bioinformatics and Computational Biology Program, University of Minnesota, Minneapolis, MN 55455, USA

**Keywords:** transcriptional noise, aging, single-cell RNA sequencing

## Abstract

Increasing stochasticity is a key feature in the aging process. At the molecular level, in addition to genome instability, a well-recognized hallmark of aging, cell-to-cell variation in gene expression was first identified in mouse hearts. With the technological breakthrough in single-cell RNA sequencing, most studies performed in recent years have demonstrated a positive correlation between cell-to-cell variation and age in human pancreatic cells, as well as mouse lymphocytes, lung cells, and muscle stem cells during senescence in vitro. This phenomenon is known as the “transcriptional noise” of aging. In addition to the increasing evidence in experimental observations, progress also has been made to better define transcriptional noise. Traditionally, transcriptional noise is measured using simple statistical measurements, such as the coefficient of variation, Fano factor, and correlation coefficient. Recently, multiple novel methods have been proposed, e.g., global coordination level analysis, to define transcriptional noise based on network analysis of gene-to-gene coordination. However, remaining challenges include a limited number of wet-lab observations, technical noise in single-cell RNA sequencing, and the lack of a standard and/or optimal data analytical measurement of transcriptional noise. Here, we review the recent technological progress, current knowledge, and challenges to better understand transcriptional noise in aging.

## 1. Introduction

**Figure 1:**
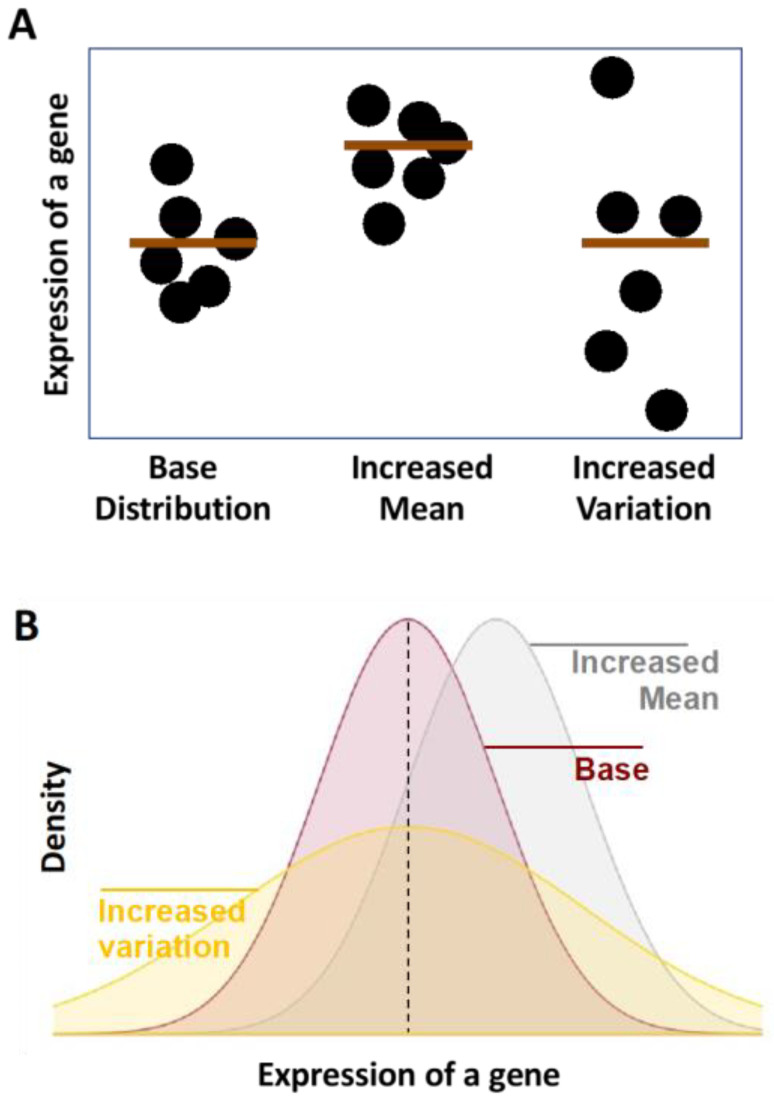
Schematic illustrations of transcriptional noise. (**A**) Each dot presents the expression level of a gene in one cell. (**B**) Distributions of expression levels of a gene across populations of cells are shown.

Age is the most significant risk factor for modern death-causing diseases, including cancers, cardiovascular disease, neurodegenerative disease, and metabolic syndromes [1,2,3,4,5,6]. As proposed in the Geroscience hypothesis, the risks of these diseases can be reduced by alleviating aging [7]. Aging, defined in brief as the age-related decline in cellular fitness, is associated with multiple hallmarks [8,9]. Of note, three of these hallmarks include the genomic, epigenomic, and proteomic aspects of macromolecular alterations. While often addressed in isolation, all three of these hallmarks appear to be increasingly interconnected [10]; in fact, it has been recently argued that the root cause of all the hallmarks is DNA damage [11,12]. Of note, the existence of age-related transcriptional alterations, which should serve as a key molecular hub connecting a network of the macromolecular hallmarks, remains largely undetermined, likely because age may have a significant effect on intra-tissue gene expression variation, but a less significant effect on mean expression levels of the entire tissue (Figure 1A,B) [13,14].

Cell-to-cell transcriptional variation within a population was first observed in strains of *Escherichia coli* that had been modified to express two alleles of a fluorescent protein in the same cells as a marker for transcription [15]. This study concluded that cell-to-cell transcriptional variation is driven by the natural stochasticity of the chemical processes in gene transcription and fluctuations in other cellular components. Using single-cell reverse transcription quantitative PCR (RT-qPCR), an age-related increase in cell-to-cell transcription variation, called “transcriptional noise”, was first observed in several housekeeping genes and cell-type specific genes of mouse cardiomyocytes [16]. Since the development of single-cell RNA sequencing (scRNA-seq) technology, more recent studies have investigated this age-related increase in noise transcriptome-wide and in more tissue types. This review discusses these studies, the various methods used to quantify transcriptional noise in aging, and remaining challenges.

## 2. Components of Transcriptional Noise

Transcriptional noise has been proposed to comprise multiple sub-components; however, the definition and exact segmentation of these components are not widely agreed upon. An early study suggested that transcriptional noise is made up of two components: intrinsic noise and extrinsic noise [15]. Intrinsic noise results from the natural stochasticity associated with biochemical reactions in the gene expression process and affects each gene independently within a cell. Extrinsic noise results from environmental factors that affect all related genes within a cell. For example, gene expression within a cell can be affected by the concentration, state, and location of certain molecules that regulate transcription. Differences in the relative quantity and activation of these key molecules between cells result in cell-to-cell transcriptional heterogeneity. However, it has been argued that it is still difficult to define the intrinsic and external noise in the context of a biological system [17]. Additionally, intrinsic and extrinsic noise are dependent on each other [18,19].

A more recent model divides transcriptional noise into three components: gene-state variables, regulatory variables, and system-state variables [18]. Gene-state variables, e.g., kinetic parameters of RNA synthesis or degradation, account for the factors directly responsible for the transcription of an individual gene. Like intrinsic noise, gene-state variables cause variation in expression independently in each gene. Regulatory variables, e.g., epigenetic factors, affect several genes at the same time; therefore, the definition of the regulatory variable component is similar to the original definition of extrinsic noise. At a larger scale, system-state variables, e.g., circadian clock and aging, are suggested to affect all genes within a cell, although the effect of system-state variables on transcriptional noise is the least understood.

## 3. Wet-Lab Methods to Quantify Transcriptional Noise

A single-cell analysis is required to discover cell-to-cell variations within a cell population or tissue. As mentioned earlier, transcriptional noise was discovered in strains of *Escherichia coli* created with two reporter genes controlled by identical regulatory sequences [15]. Both genes encoded the same green fluorescent protein, but one gene expressed a cyan allele (CFP), and the other expressed a yellow allele (YFP). To measure possible effects of transcriptional noise, cells were photographed, and the fluorescent intensity of each cell was quantified. Both intrinsic and extrinsic noise were determined by plotting the CFP versus YFP intensities of the same cells.

Quantifying transcription at the single-cell level has been technically challenging. Before the development of single-cell expression quantification methods, transcriptional noise had been indirectly discovered as early as 2005 in monozygotic twins [20]. Gene expression from old and young monozygotic twins was analyzed using microarrays. A pair of 50-year-old twins had approximately four times as many differentially expressed genes between them, as compared with the number of differentially expressed genes found in a pair of 3-year-old twins [20]. Interestingly, the older twins had not only the highest number of overexpressed genes, but also the most severe epigenetic changes on DNA methylation and histone modifications. This finding suggests there may be a possible link between transcriptional instability and epigenetic instability.

Single-cell RT-qPCR and scRNA-seq are the most direct methods used to detect transcriptional noise [16]. With the development of multiple commercially available kits, such as SMART-Seq2 and Chromium, scRNA-seq has become the most popular method and has been extensively reviewed in recent literature [21]. However, it is worth noting that scRNA-seq data suffer from a considerable amount of data dropout; transcribed genes, especially those expressed at a low level, may not be detected in a portion of cells due to the technical limitations of this method. The inability to overcome these technical limitations creates a significant challenge in distinguishing real biological variation from the technical noise. Still, most of the discoveries on age-related transcriptional noise have been made using these two methods and are discussed in the section below.

## 4. Current Knowledge about Transcriptional Noise in Aging

The relationship between transcriptional noise and aging was first discovered in mouse cardiomyocytes using single-cell RT-qPCR in 2006 [16]. This study posed a key question: “Is transcriptional instability a universal hallmark of aging?” To answer this question, studies would need to definitively identify transcriptional noise, and then determine if an age-related increase in transcriptional noise is universal across cell types and species. However, single-cell RT-qPCR can only be used to quantify a limited number of genes at a time and assessing the universality of transcriptional noise was very difficult. It was not until the late 2010s that scRNA-seq capable of quantifying nearly the entire transcriptome of individual cells has now paved the way for future studies on this topic.

Most papers on transcriptional noise have studied mice using scRNA-seq and have shown an increase in transcriptional noise with the age of the various cell types tested (Table 1), including T lymphocytes [14], dermal fibroblasts [22], lung cells [23,24], kidney cells [24], spleen cells [24], muscle stem cells [25], and hematopoietic stem cells [26]. One of the strongest pieces of evidence that transcriptional noise affects most of the cell types is found in a cell atlas describing aging mouse lungs [23]. This atlas contains single-cell transcriptome profiles of 30 lung cell types in young (3 months of age) and old (24 months of age) mice and indicate that about half of the cell types exhibit a significant increase in transcriptional noise with age. Based on the Tabular Muris Senis [27], a dataset of over 350,000 cells from 23 tissues and organs, a recent study found that there is a significant overlap in genes and their pathways with elevated transcriptional noise in different tissue types and cell types with age, suggesting that changes in transcriptional noise occur in a gene-specific manner rather than on a cell-type or tissue-type basis [28]. Of note, while the results from these studies suggest transcriptional noise is conserved across many cell types, only a limited number of cell types have been examined thus far, and a substantial proportion of the cell types examined showed no increase in transcriptional noise with age (see below and Table 1).

Even fewer studies have examined whether transcriptional noise is conserved in other species (Table 1). The only cross-species comparison that we have found was performed using T cells of two inbred mouse sub-species, *Mus musculus domesticus* and *Mus musculus castaneus*, which have been separated by a minimum of a million years of genetic divergence [14]. The results from both sub-species showed statistically significant increases in transcriptional noise in old subjects as compared with transcriptional noise in young mice from the same sub-species. In addition to studies in mice, studies conducted on human pancreatic cells [29], human senescent cell lines [30], and drosophila brain cells [26] also have shown increased transcriptional noise with age.

Although the results of several studies found an increase of transcriptional noise with age, there are results from other studies that did not confirm this phenomenon (Table 1). An early study on mouse hematopoietic stem cells, granulocytes, naïve T, and naïve B cells found that these constantly renewing cells did not exhibit a significant increase in transcriptional noise [31]. This study used single-cell RT-qPCR to quantify expression of six specific genes, although, the small number of genes quantified may lower the strength of this claim. However, a more recent study performed scRNA-seq on approximately 37,000 mouse brain cells from 25 cell types and did not find a significant increase in transcriptional noise with age [32]. A cell atlas of aging drosophila brain analyzed 157,000 cells from 30 cell types and found no change in transcriptional noise with age [33].

Interestingly, a recent study reanalyzed a small subset of the drosophila brain cells mentioned above [33], as well as murine hematopoietic stem cells [34,35,36,37], murine T lymphocytes [14], human pancreatic cells [29], and human carcinoma cell lines [38], and discovered an age-related increase in transcriptional noise using a newly developed global coordination level (GCL) analysis [26]. Additionally, Ibáñez-Sole et al. re-examined seven previous studies on transcriptional noise using another newly developed tool known as Scallop [39]. Although six of these studies reported an age-related increase in transcriptional noise, Scallop analysis confirmed the results of only two studies, and failed to detect an increase in transcriptional noise in the remaining studies. Overall, the contradictory results of the studies above may be due, in large part, to the different quantitative, statistical, or informatic measurements (or “definitions”) of transcriptional noise used. Unfortunately, it is difficult to resolve these discrepancies, because it is still unclear which, if any, method can accurately measure transcriptional noise, and different noise definitions quantify different aspects of variation. Below, we classify and review the definitions of transcriptional noise into two categories: classical measurements of data variation, which have been adapted for transcriptional noise, and novel measurements, which have been designed specifically for transcriptional noise.

**Table 1:** A summary of studies about transcriptional noise in aging.

Species	Cell Type	Increase with Age?	Study	Quantification Method	Year
Mouse	Cardiomyocytes	Yes	Bahar et al. [16]	Variance	2006
	Hematopoietic Stem Cells	No	Warren et al. [31]	CoV	2007
		Yes	Levy et al. [26]	GCL	2020
	Multiple Lymphocyte Types	No	Warren et al. [31]	CoV	2007
		No	Ibáñez-Sole et al. [39]	Decibel, Scallop	2022
		Yes	Martinez-Jimenez et al. [14]	BASiCS	2017
	Multiple Lung Cell Types	Yes	Angelidis et al. [23]	Correlation	2019
		No	Ibáñez-Sole et al. [39]	Decibel, Scallop	2022
		Yes	Kimmel et al. [24]	Overdispersion, Correlation	2019
	Granulocytes	No	Warren et al. [31]	CoV	2007
	Muscle Stem Cells	Yes	Hernando-Herraez et al. [25]	Correlation	2019
	Liver Cells	Yes	de Jong et al. [18]	GAMLSS	2019
	Hematopoietic Multipotent Progenitors	Yes	Levy et al. [26]	GCL	2020
	Multiple Brain Cell Types	No	Ximerakis et al. [32]	CV	2019
		No	Ibáñez-Sole et al. [39]	Decibel, Scallop	2022
	Multiple Kidney Cell Types	Yes	Kimmel et al. [24]	Overdispersion, Correlation	2019
		No	Ibáñez-Sole et al. [39]	Decibel, Scallop	2022
	Multiple Spleen Cell Types	Yes	Kimmel et al. [24]	Overdispersion, Correlation	2019
		No	Ibáñez-Sole et al. [39]	Decibel, Scallop	2022
	Dermal Fibroblasts	Yes	Salzer et al. [22]	Clustering	2018
		Yes	Ibáñez-Sole et al. [39]	Decibel, Scallop	2022
	23 Tissues and Organs	Yes	Marti et al. [28]	TINA	In preprint
Drosophila	Multiple Brain Cell Types	No	Davie et al. [33]	Clustering, Trajectory analysis	2018
		Yes	Levy et al. [26]	GCL	2020
Human	Islet Endocrine Cells	Yes	Enge et al. [29]	Correlation	2017
		Yes	Ibáñez-Sole et al. [39]	Decibel, Scallop	2022
	Fibroblasts	Yes	Wiley et al. [31]	Variance, Correlation	2017

## 5. Defining Transcriptional Noise Using Classical Measurements of Data Variation

Quantifying transcriptional noise is difficult, not only because total variation in gene expression can be small, but also because it is difficult to distinguish real biological noise from technical noise, as mentioned above. Therefore, to accurately quantify transcriptional noise, a measurement must be precise enough to detect subtle changes in variation and robust enough to allow the separation of real variation in gene expression from technical noise. As of the writing of this paper, there is no universally accepted standard measurement of transcriptional noise, although a wide variety of statistical and informatics definitions have been used to analyze the results of various studies.

The most common of these measurements are the coefficient of variation (CoV) and Fano factor (FF). CoV is defined as the ratio of the standard deviation (σ) to the mean (μ; Equation (1)). While standard deviation is often used to measure the spread of a single dataset, CoV often is used to compare the variations between two different datasets. FF is defined as the ratio of the square of the standard deviation to the mean (Equation (2)), and is subsequently more sensitive to outliers than CoV, while CoV is the used more often [40,41,42]. Although both CoV and FF measure transcriptional noise, these methods also have significant limitations. CoV and FF can be used to quantify total transcriptional noise by first measuring the variation of every gene across a cell population and then taking the average of these values to find the variation in the “average” gene of a cell. However, it still is not known whether the observed age-related increase in transcriptional noise is driven by most of the genes in the genome or by only a few genes that exhibit a greater variability. If only a small number of genes show an increase in variability with age, then quantifying transcriptional noise as the average variation of every gene is an inappropriate way to evaluate these data. While this limitation could be overcome by measuring and averaging the variation in only a specific subset of genes, more studies need to be undertaken to validate this approach. Additionally, both methods contain intrinsic limitations, as their values depend on data dimensionality and distribution. Ignoring these limitations can lead to an incorrect interpretation of the results, which will significantly affect, and potentially distort, the conclusions of studies in the primary literature of this emerging field that use CoV or FF as a measurement of variation [42,43].

(1) $$\mathrm{CoV}=\frac{\sigma }{\mu }$$

(2) $$\mathrm{FF}=\frac{\sigma^{2}}{\mu }$$

The correlation coefficient between the transcriptomes of a pair of cells or between the transcriptomes of a cell and a defined “average” cell of a population is another widely used measurement [23,25,29]. There are different methods that can be applied to quantify correlation coefficient. For example, the Pearson’s product moment correlation coefficient (or “Pearson correlation”) evaluates linear relationships and is best suited for datasets where both variables are normally distributed, while the Spearman’s rank correlation coefficient (or “Spearman correlation”) evaluates monotonic relationships typically used for non-normally distributed variables [44]. These two correlation measurements result in small, but significant, differences when applied to the same datasetč therefore, the choice of method can affect the interpretation of data from a study [44].

Several studies have used a wide variety of methods to “trim” the list of genes from the entire transcriptome to be used as input in correlation analysis. Only selected genes, instead of every gene in the transcriptome, were used to calculate correlation coefficients. Although limiting the number of genes to be analyzed offers certain advantages, e.g., reducing computational effort and cleaning up technical noise, the number and choice of genes differ between studies and remain somewhat arbitrary. For example, the 500 most variable genes were used in one study [25], while the 500 most highly expressed genes were used in two other studies [23,29].

Other methods also can be applied to examine transcriptional noise, such as entropy [45] and generalized additive model for location, scale, and shape (GAMLSS) [18,46]. However, as mentioned above, variations in quantification methods are significant enough to yield conflicting results. Importantly, it is unclear if differences in trimming criteria mentioned above would also contribute to the conflicting results.

## 6. Novel Definitions of Transcriptional Noise

In addition to the implementation of existing measurements of data variation, novel measurements have been developed specifically for transcriptional noise. These include Bayesian analysis of single-cell sequencing (BASiCS) [47], the method of Isildak et al. (which we call residual-based correlation; RBC) [48], global coordination level (GCL) [26,49], Decibel [39], Scallop [39], and the method of Marti et al. (which we call technically induced noise approximation; TINA) [28]. Because these methods involve more comprehensive definitions than CoV and FF, the reader can refer to the original papers for their mathematical or statistical equations.

BASiCS is an integrated Bayesian hierarchal model, developed for analyzing single-cell RNA sequencing [47]. This method can distinguish real variation from technical noise by leveraging information gained from artificially spiked genes that have been introduced into each cell. Once the technical noise has been filtered out, gene expression variation is estimated using gene-specific over-dispersion parameters that measure the residual variance in each gene [47,50]. While multiple studies have used BASiCS for various types of data analysis [51,52,53], only a single study has focused on measuring the age-related increase in transcriptional noise using this method [14].

RBC quantifies transcriptional noise while also considering non-random expression changes with age [48]. First, the RBC method utilizes a linear regression correlating each individual gene and age. Then, the unexplained residuals of the regression model, which reflect transcriptional noise, are correlated again with age using a Spearman correlation. A highly positive Spearman correlation coefficient indicates a strong age-related increase in transcriptional noise. While most other methods quantify the transcriptional noise on a dataset-wide basis, RBC measures the variability of each gene individually, making it uniquely suited for identifying the individual genes in which variability changes with age. While RBC is a more sophisticated extension of past correlation methods, its feasibility to measure transcriptional noise in scRNA-seq data is unknown, as thus far, it has only been used to assess inter-individual heterogeneity in gene expression using microarray data.

GCL analysis measures the dependency between random subsets of genes within a single cell as the global level of coordination between genes in a cell [26]. First, the genes are randomly divided into two equally sized subsets. Then, the dependency between these subsets is measured using bias-corrected distance correlation (bcdCorr), which, in theory, can be replaced by a different high-dimensionality dependency measurement technique. By repeating these two processes a sufficient number of times, the dependency levels between many different gene sets can be measured and a robust GCL value of a single cell can be determined by averaging the calculated dependency levels. A higher GCL value indicates lower transcriptional noise. While GCL analysis is a promising new method that incorporates the detection of an age-related increase in transcriptional noise in a wide variety of different species and cell types, pre-filtering the input of scRNA-seq data has been shown to be critical because “outlier” cells and cell clustering can significantly impact its results [49].

Decibel combines results of four existing methods to measure transcriptional noise [39]: (i) biological variation over technical variation [29], (ii) Euclidean distance between each cell and a population’s “average” cell [29], (iii) Euclidean distance using a subset of invariant genes [29], and (iv) GCL [26]. The first method measures transcriptional noise by first measuring total variation using the Pearson correlations between the transcriptomes of cells and their “average” cell, and then removes technical variation estimated from ERCC spike-ins. The second method measures the Euclidean distance between the transcriptomes of cells and their “average” cell. The third method is an extension of the second method, but only a subset of genes from the entire transcriptome is used. As explained above, there are multiple methods for “trimming” gene lists from a dataset. Decibel uses an approach outlined in Enge et al. where genes are split into equal sized bins based on their expression and the bins containing the most expressed and least expressed genes are discarded. In the remaining bin, the top 10% most variable genes, as measured by CoV, are used for analysis [29]. Decibel does not further aggregate the results of these models to derive a single score, but keeps all the values of different models [39].

Scallop assigns each cell a membership score, reflecting the strength of the cell’s association with a cell cluster [39]. Higher strength suggests lower transcriptional noise of the cell. Membership scores are calculated in three steps: bootstrapping, cluster relabeling, and scoring. Bootstrapping is composed of three steps: first the total data is resampled into a subset of cells (e.g., 95% of the total); second, the subset of cells is clustered based on a predetermined clustering algorithm (e.g., Leiden [54]); and finally, the two steps above are repeated multiple times (i.e., iterations). Of note, the clustering results of different iterations are not comparable, as they are generated using slightly different sets of cells; therefore, cluster relabeling is needed to compare cluster annotations across iterations. In cluster relabeling, each cluster in each iteration is relabeled to a reference cluster such that “cluster X” refers to the same cluster in every bootstrap iteration. This is achieved by comparing each cluster in each iteration to a reference clustering; clusters that greatly overlap are assumed to be a good match and the original cluster is relabeled based on its closest match in the reference clustering. To generate a reference clustering, the clustering algorithm used in bootstrapping above is applied to the original dataset without any cells removed. Finally, each cell is given a membership score between 0 and 1, which represents the fraction of iterations in which a cell was assigned to the same cluster. This method assumes that transcriptionally noisy cells will be assigned inconsistent clusters across bootstrap iterations and, therefore, receive a low membership score, while transcriptionally stable cells will be predominantly assigned to a single cluster and have relatively higher membership scores.

Finally, TINA attempts to separate true biological noise from technically induced noise when analyzing scRNA-seq data [28]. TINA assumes technical noise is induced in four distinct steps: variations per cell in mRNA capture efficiency, variations per cell in the number of reverse transcribed mRNA molecules, the probability that a given mRNA molecule is amplified during each PCR step, and variations per cell in the PCR efficiency. This quantitative model deconvolves the count per million (cpm) of a given gene into an estimate of the mean number cDNA molecules (biological noise) and their noise factor (technical noise) using only four input parameters to account for technically induced noise: relative capture efficiency, PCR gain, PCR noise, and a noise factor that represents well-to-well variation. Like RBC, TINA measures the transcriptional noise of a given gene across all cells, instead of the noise across an entire dataset or of a given cell.

## 7. Conclusions

Although first proposed 15 years ago, transcriptional noise in relation to aging has been examined in only a few studies. Results from most studies have suggested that transcriptional noise is likely a conserved hallmark of aging, but the findings of these studies have been limited to only a few cell types, primarily from mice. The lack of agreement on the analytical measurement of transcriptional noise reflects both the comprehensiveness in considering technical noise and the effect of cell clusters in the scRNA-seq data, as well as the possible difference in the transcriptional noise of different genes and genetic pathways. The cause and consequence of transcriptional noise in aging is another unexplored territory. Although it is conceivable that the instability at the transcriptional level can be caused by instabilities at the DNA level, e.g., de novo mutations [13] and epigenetic instabilities [25,55], a single-cell multi-omics approach will have to be developed in order to evaluate the causal relationship of transcriptional noise, other macromolecular instabilities, and aging within the same single cells.
